# The Changing Care of Older Adults With Bipolar Disorder: A Narrative Analysis

**DOI:** 10.1177/10497323241263043

**Published:** 2024-07-30

**Authors:** Aaron Warner, Jasper Palmier-Claus, Carol Holland, Elizabeth Tyler, Verity Rhodes, Geoff Settle, Fiona Lobban

**Affiliations:** 1 Institute of Population Health, 4591 University of Liverpool, Liverpool, UK; 2 Division of Health Research, Spectrum Centre for Mental Health Research, Lancaster, UK; 3 Lancashire & South Cumbria NHS Foundation Trust, Lancashire, UK; 4 Division of Health Research, Centre for Aging Research, Lancaster, UK; 5 Division of Psychology & Mental Health, 5292 University of Manchester, Manchester, UK; 6 NIHR Applied Research Collaboration North West Coast, UK

**Keywords:** bipolar disorder, ageing, mania, healthcare, narrative, qualitative

## Abstract

Older adults with bipolar disorder experience distinct challenges compared to younger age groups with bipolar disorder. They potentially require adaptations to the care they receive. This study aimed to explore experiences of care and changing care needs in older adults with bipolar disorder. People with bipolar disorder (aged ≥60) were recruited through three NHS Trusts in the North West of England, charity organisations, a confidential university participant database, and social media. Participants completed single time-point biographical narrative interviews, which were analysed using narrative analysis. Sixteen participants’ accounts led to the creation of four themes: (1) ‘Navigating the disruption caused by diagnosis’; (2) ‘The removal of services that provided hope’; (3) ‘Later life: We are on our own now’; and (4) ‘Changing care needs in later life: We still need support’. The care needs of older adults with bipolar disorder appear to change over time, and services often fail to offer adequate, tailored care for this group at present. Current support requires adaptation to be effective and appropriate and to enable this group to age well in later life.

## Introduction

Bipolar disorder is characterised by recurrent episodes of elevated mood (mania and hypomania), alongside periods of depression and disruptions to thought and behaviour that can affect quality of life (American Psychiatric Association, 2013). Bipolar disorder is often categorised into two main subgroups, bipolar I and bipolar II (American Psychiatric Association, 2013). To meet the criteria for bipolar I, individuals must have experienced an episode of mania for 1 week or longer (Anderson et al., 2012). Depressive episodes are common for people who receive this diagnosis, although they are not a requirement (Grande et al., 2016). For bipolar II, individuals must have experienced at least one hypomanic episode and one major depressive episode (lasting for 2 weeks or longer) (Anderson et al., 2012). Bipolar disorder is believed to affect up to 1% of the population worldwide (Dautzenberg et al., 2016). At present, approximately 10–25% of all individuals diagnosed with bipolar disorder are aged 60 or over (Dautzenberg et al., 2016). These numbers are expected to increase dramatically by 2030 due to the ageing of the total population and improved awareness of bipolar disorder (Depp & Jeste, 2004). Research highlights that bipolar disorder in older adults is potentially more complex to treat due to an increased number of physical health comorbidities (Lala & Sajatovic, 2012; Warner et al., 2023), accelerated cognitive decline (Schouws et al., 2009), and less social support when compared to younger age groups with bipolar disorder (Beyer et al., 2003). Consequently, more research is needed to explore the changing care needs of older adults with bipolar disorder to enable us to improve the care and support available for this group.

According to the life course perspective (Dannefer & Settersten, 2010), ageing is a lifelong, interactive process that is continuously evolving. It suggests that people’s ability to age well can be influenced by inequalities experienced throughout their lives (Dannefer & Settersten, 2010). Ageing with bipolar disorder may, therefore, be particularly challenging as it is associated with several difficulties and inequalities that appear to affect this group’s wellbeing in later life (Sajatovic et al., 2015). This is highlighted in existing literature, which suggests that older adults with bipolar disorder experience distinct psychological and emotional challenges compared to younger age groups with bipolar disorder (Clifton et al., 2013; Depp & Jeste, 2004). Nivoli et al. (2014) found that bipolar disorder in older adults is associated with longer manic and depressive episodes and shorter inter-episode intervals compared to younger populations. Research also indicates that people with late-onset bipolar disorder experience more manic episodes and more severe cognitive impairment than those with early onset (Schouws et al., 2009). Additionally, Goldstein et al. (2006) determined that older adults with bipolar disorder experience a significantly higher prevalence of psychiatric comorbidities such as alcohol use disorder (38.1% vs. 14.4%), panic disorder (19% vs. 2.5%), and generalised anxiety disorder (20.5% vs. 2.5%), compared to age-matched controls without bipolar disorder. Consequently, older adults with bipolar disorder potentially face different challenges compared to younger age groups with bipolar disorder and the general ageing population, meaning they require adaptations to the care they receive (Sajatovic et al., 2015).

The National Institute for Health and Care Excellence (NICE, 2014) recommends the same treatments to older adults with bipolar disorder as those for younger age groups. At present, the long-term use of mood-stabilising medication remains the most common treatment (Morlet et al., 2019). Literature suggests that the long-term use of certain medications, such as lithium, can improve outcomes and also decrease the risk of Alzheimer’s disease (Nunes et al., 2007). However, there is also an indication that the long-term use of mood-stabilising medication can increase the risk of cognitive difficulties (Pachet & Wisniewski, 2003) and physical health difficulties such as diabetes, weight gain, and renal failure in later life (Lala & Sajatovic, 2012; Sajatovic et al., 2022). Authors have argued that mood-stabilising medications have concerning side effects in the long term (Morlet et al., 2019). They are also reported to be insufficient to address the changing physical, psychological, and social care needs of older adults with bipolar disorder (Morlet et al., 2019). Additionally, older adults with bipolar disorder may have more passive coping styles than the general population and are more accepting of their difficulties (Schouws et al., 2015). Whilst this appears to enable older adults with bipolar disorder to adapt and complete new activities, it also suggests that this group may become more resigned to their challenges and attempt to cope independently rather than seeking support from services (Schows et al., 2015). When older adults with bipolar disorder do seek help, they often receive inadequate treatments that fail to meet their changing care needs (Dautzenberg et al., 2016). This can result in this group disengaging from services completely, leaving their needs unmet and potentially contributing to reduced quality of life and poor clinical outcomes (Dautzenberg et al., 2016).

Existing qualitative research has identified that individuals with bipolar disorder value care that involves proactive staff, meaningful connections with professionals, and feelings of safety and sociability (Skelly et al., 2013). However, there is insufficient qualitative research exploring the changing care needs of people with bipolar disorder as they enter later life. Consequently, our knowledge about how best to support this group as they age is limited and may lead to insufficient and ineffective care (Sajatovic et al., 2015). One qualitative study using thematic analysis and photo elicitation explored what it means to age well with bipolar disorder. The results suggested that older adults with bipolar disorder experience unique challenges that require services to adapt to meet their needs (Warner et al., 2024). This study aims to build upon this research and address an important gap within the literature by qualitatively exploring the changing care and care needs of older adults with bipolar disorder in later life. This could identify necessary adaptations that might improve the support offered to this group and enhance their quality of life as they age. The study uses biographical narrative interviews (Wengraf, 2001) to capture the experiences of older adults with bipolar disorder since they first tried to seek help for bipolar disorder or first came into contact with mental health services until the time of the interview in later life. This approach provides the opportunity to identify how and why peoples’ care needs change over time. It also helps to consider how services might adapt and tailor support, with the ultimate goal of improving wellbeing and tackling health inequalities.

## Methods

### Research Aims

To explore how the care and care needs of older adults with bipolar disorder change in later life.

### Design

The epistemological position of the researcher influences the research design (Roots, 2007). In this study, the researchers adopted a constructionist position emphasising that participants’ stories were created collaboratively between the participant and researcher (Esin et al., 2014). This position considered how each participant’s unique personal, social, and cultural experiences shaped the telling of their story (Esin et al., 2014). The researchers’ position resulted in aims that were focused on making sense of participants’ experiences, and therefore, qualitative methods were appropriate (Hammarberg et al., 2016). The lead author completed single time-point biographical narrative interviews (Wengraf, 2001) with participants, which were analysed using narrative analysis.

### Participants and Recruitment

Participants were purposively sampled through (1) community-based mental health services within three NHS Trusts in the North West of England, (2) a confidential university-based participant database consisting of people living with mental health difficulties who previously consented to be contacted about research projects, (3) advertising on social media, and (4) UK-based mental health charity organisations, such as Bipolar Scotland. The inclusion criteria for the study were (1) aged ≥60; (2) a self-reported diagnosis of bipolar I or II disorder from a healthcare professional during their life; and (3) reporting experiences consistent with bipolar disorder for 10 or more years to ensure that the researcher could understand how and why their care and care needs changed over time. Capacity to provide informed consent was assessed by the interviewer or by a clinician who worked directly with the participant if they were recruited from NHS services. All participants were required to have adequate English language skills to complete the interview. Exclusion criteria included a known moderate to severe cognitive impairment or learning disability, any imminent current risk to the self or others, and current inpatient or crisis service input.

### Data Collection

Participants provided informed written or audio consent to take part after reading an information sheet detailing the aims of the study and were able to ask the researchers questions prior to their participation. They then completed single time-point biographical narrative interviews (Wengraf, 2001) with the lead author (AW). All interviews were completed either online using Microsoft Teams (*n* = 10), via telephone (*n* = 3), in the participant’s home (*n* = 2), or at an NHS base (*n* = 1). Interviews ranged from 50 min to 130 min in duration. These interviews were composed of three stages. In stage 1, the interviewer aimed to capture participants’ experiences of care and their changing care needs over time by asking the question, ‘Please could you tell me about your experiences of care and support since you first tried to get help for bipolar disorder, or first came into contact with mental health services, up until now?’. During this stage, the interviewer took the role of an interested listener and allowed participants to tell their stories without interruption. The interviewers used mainly non-verbal cues to offer encouragement during this stage, as recommended by Rosenthal (1993). In stage 2, the interviewer waited for the participant to voluntarily break off from telling their story before asking narrative-informed questions about the biographical content that the participant covered during the initial telling of their story (e.g., what happened after that, what care did you receive following that?). Following stages 1 and 2, the interviewer offered participants the opportunity to have a break before asking questions about areas that were not covered during stages 1 or 2 but seemed to be important in answering the research question comprehensively (e.g., can you tell me what you feel your key care needs are at present and why?). Questions for stage 3 of the interview were developed by the research team alongside older adult public advisors with experience of mental health difficulties. Finally, the interviewer asked whether participants wanted to add anything before concluding the interview. Participants provided demographic information for descriptive purposes.

### Data Analysis

The authors analysed the interview transcripts using narrative analysis. This approach allows researchers to make sense of participants’ stories and considers the role that culture, relationships, and language have in shaping them (Wertz, 2011). Narrative analysis is also appropriate for the current study as it can identify important transitions and events that have occurred in participants’ lives (Wertz, 2011). This helps to provide insight into their past experiences, present behaviours, and future desires in relation to their care (Esin, 2011). Leading figures in narrative analysis stress that there is no one way to complete a narrative analysis (Esin, 2011; Frost, 2009). Researchers are encouraged to be imaginative and innovative while remaining reflexive and ensuring rigour in the analytical process (Frost, 2009).

The analysis comprised the following steps. The lead author (AW) transcribed all interviews verbatim before reading and re-reading participants’ interviews to immerse himself in the data (Crossley, 2000). Narrative concepts, such as the tone in which each participant’s story was told, were noted during this initial process (Crossley, 2000). This helped highlight the participant’s feelings towards the topic (Smith, 2016). The lead author then worked with one interview at a time to generate initial codes and identify recurrent topics and stories within participants’ narratives (Anderson & Kirkpatrick, 2016). During this process, a one-page summary of each participant’s narrative was developed to highlight key events within their interview (Jovchelovitch & Bauer, 2000). They then worked to develop key themes that highlighted similarities and differences in the telling of participants’ stories (Esin, 2011). The themes aimed to identify broader patterns of meaning across the dataset and portray key events, transitions, and changes in the care and care needs of older adults with bipolar disorder. Finally, the themes were outlined in a way that told a coherent story of the experiences of care and support (Anderson & Kirkpatrick, 2016).

Throughout the analytical process, participant narratives were explored on the personal, interpersonal, positional, and ideological levels outlined by Murray (2000). On the personal level, the researchers examined how participants’ stories portrayed how they viewed the world and their sense of self and considered what function the telling of their stories had for participants (Murray, 2000). On the interpersonal level, the researchers assessed their role in shaping the participants’ narrative accounts. On the positional level, the researchers considered the differences in social position which existed before the interaction between the participant and interviewer (Murray, 2000), such as social power and how this may have influenced the interview process and subsequent narrative. Finally, at the ideological level, the role that societal beliefs, systems, and ideologies might have had in developing the narrative told by the participant were considered (Doise & Mapstone, 1986). This included issues such as stigma surrounding mental health and ageing and the impact of seeking support from the National Health Service (NHS) or other relevant services. These levels were considered throughout the analytical process and used to provide an in-depth narrative account of participants’ experiences of care and their changing care needs as they aged with bipolar disorder.

### Data Sufficiency

To determine whether sufficient data had been collected, the researchers reviewed the aims of the study, the richness of the data, and the method of analysis (Malterud et al., 2016). To ensure that the decision to stop collecting data was informed by multiple perspectives, the research team considered whether data was sufficient to address the aims of the study alongside public advisors with lived experiences of ageing with mental health difficulties. Whilst decisions surrounding data sufficiency are inherently subjective, these considerations ensured the process of determining when to conclude data collection was as reflexive and rigorous as possible.

### Reflexivity and Enhancing Rigour

The lead author for this study was a PhD student at a UK university who also had experience working alongside older adults experiencing mental health difficulties within the NHS. These experiences potentially influenced how the study was designed, conducted, and analysed. The research team took several steps to ensure transparency and rigour at all stages of the research process (Jootun et al., 2009). Before data collection, AW completed a positionality statement that identified their philosophical position, gender, class, and personal experiences and considered how these might influence their perceptions of the data (Holmes, 2020). AW also engaged in regular supervision with the research team, which offered the opportunity to discuss any challenges and receive feedback on the study design, data collection, and analysis at all stages. Three members of the research team were qualified clinical psychologists (FL, JPC, and ET), one was a Professor in Ageing (CH), and two were older adult public advisors with lived experiences of mental health difficulties (VR and GS). This meant that there were multiple perspectives from people with different backgrounds available to the researcher at all stages of the research. Feedback from the research team also helped to identify potential biases that may have influenced the way that the research was completed. It ensured that analytical interpretations were as consistent with the experiences of participants as possible (Lincoln & Guba, 1986). Furthermore, AW kept a reflexive diary throughout the research process, highlighting any methodological or personal challenges that may have influenced how the research was conducted to ensure transparency in decision-making (Watanabe, 2017). Finally, an audit trail consisting of observational notes, raw data, and reflections following interviews was kept by the researcher to aid reflection on decisions that informed interpretation of the data as recommended by Johnson & Colleagues (2020).

### Ethics

This study received NHS Health Research Authority approval (REC reference: 21/LO/0405). It also received approval from three NHS Trusts that allowed the research team to recruit participants from their services. All participants had to provide written or audio-recorded verbal consent before participating in the study.

## Results

Eighteen older adults with bipolar disorder were invited to take part in the study. Two declined as they did not wish to take part at that time, resulting in sixteen biographical narrative interviews. Seven participants were recruited from a confidential university research database, four participants were from NHS services, four were from mental health charities, and one was from social media. The mean age of participants was 66.1 (60–75; SD 4.9), the majority were female (69% vs. 31% males), and most participants reported a diagnosis of bipolar disorder I (88%). The sample was also predominantly White British (50%), with 5% identifying as White Other, 6% Black British, 6% Indian, and 6% Asian British (see Table 1 for participant characteristics). All participants were given pseudonyms to maintain their confidentiality and anonymity.

**Table 1.** Participant Characteristics.

Demographics (*n* = 16)	Total
Age, *mean* (range, SD)	66.1 (60–75, 4.9)
Gender (*n*, %)	
Female	11 (69%)
Male	5 (31%)
Ethnicity (*n*, %)	
White British	8 (50%)
Black British	1 (6%)
White Other	5 (32%)
Indian	1 (6%)
Asian British	1 (6%)
Diagnosis (*n*, %)	
Bipolar disorder I	14 (88%)
Bipolar disorder II	2 (12%)
Employment status (*n*, %)	
Retired	6 (37.5%)
Unemployed	6 (37.5%)
Employed	4 (25%)
Living status (*n*, %)	
Alone	9 (56%)
With spouse	5 (32%)
With children	1 (6%)
Prefer not to say	1 (6%)
Education (highest level) (*n*, %)	
No qualifications	2 (12%)
Secondary	3 (19%)
A/O level	1 (6%)
Undergraduate degree	7 (44%)
Postgraduate degree	3 (19%)
Number of hospitalisations, *mean* (range, SD)	2.4 (0–10, 2.6)
Age when diagnosed, *mean* (range, SD)	33.3 (18–54, 13.5)
Place recruited from (*n*, %)	
NHS service	4 (25%)
Research participant database	7 (44%)
Social media	1 (6%)
Charity organisation	4 (25%)

**Figure 1.**
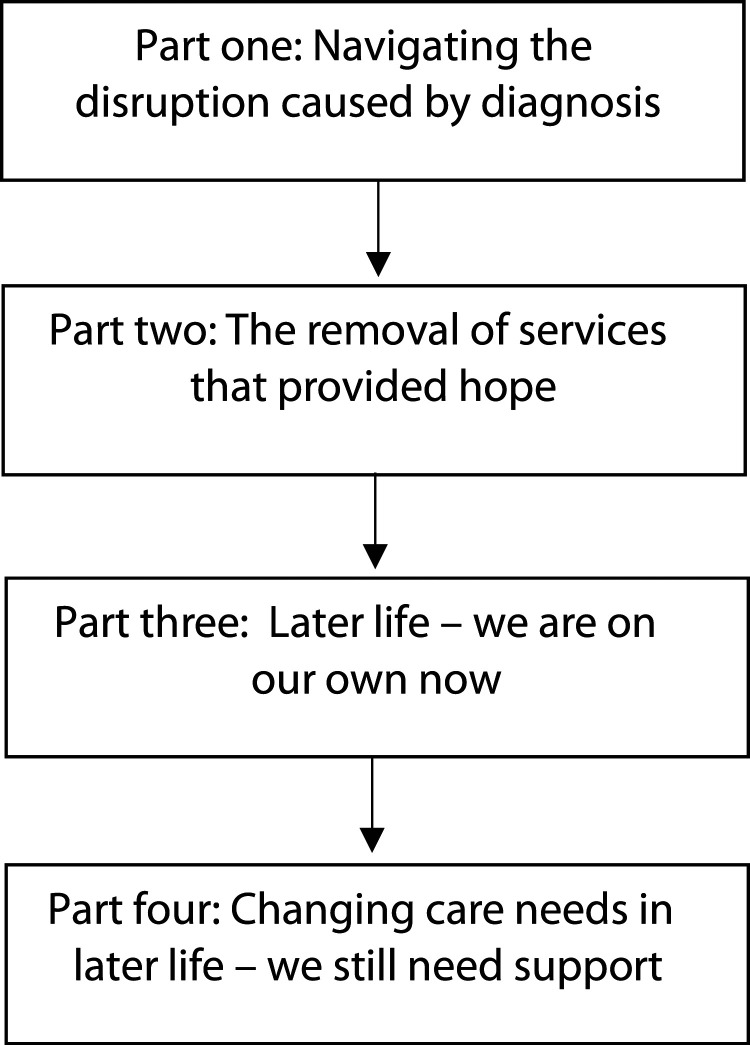
Parts of participants’ narratives.

Participants’ stories highlighted four key phases that provided insight into the changing care and care needs of older adults with bipolar disorder over their lifetime. Stories have been split into parts as recommended by Wilson et al. (2015). Part 1 focused on participants’ initial confusion and fear after being diagnosed with bipolar disorder and their subsequent attempts to access support. Part 2 highlighted how community care and peer support provided brief hope and safety following a diagnosis before this disappeared, leaving participants disappointed. Part 3 illuminated participants’ frustrations at being unable to access previously beneficial support in later life. This led to some feeling as though they were managing bipolar disorder alone, whilst others disengaged with services completely to avoid iatrogenic stress as they aged. Finally, in part 4, participants described feeling that their care needs had changed in later life and suggested that their support required adaptation to remain effective as they aged. Overall, participants’ narratives portrayed a story that displayed a disconnect between the care they received in later life and the care required to meet their changing needs. Each part of the narrative is explored in more detail below (see Figure 1 and Table 2).

**Table 2.** Additional Supporting Extracts for All Themes.

Theme	Example Quotes	Source
1: Navigating the disruption caused by diagnosis	*When I was diagnosed, I was excited to tell people that someone had told me what was wrong. After that, I noticed that people started to withdraw because they thought I was odd and I had a mental label. I became very lonely after that.* *When I was first diagnosed, I wasn’t offered any care or support, just medication, no care at all. It was scary.* *I was terrified to tell anyone that I had a diagnosis of bipolar disorder. They’d think of me differently, and they wouldn’t understand. I didn’t feel like I could talk to anyone about it.*	David Linda Debbie
2: The removal of services that provided hope	*Things were alright. While there was support for people in the community, they started discharging people into the community without support or a CPN. After that, there wasn’t any support, and it fell apart.* *When I was back in the community, I was given a CPN who would help me figure out how to spend my time. It was helpful, and I think it’s quite astonishing that it doesn’t happen anymore. This is the trouble.* *Respite services were so helpful, but then they just removed them. Nothing like that is offered in my area anymore.*	Mandy Amy Debbie
3: Later life – We are on our own now	*I was discharged after a fairly lengthy period in hospital. I was amazed that there was hardly any follow-up. I’ve just been left alone.* *The only way that I have been able to access care is to find a private doctor. I am lucky because I have the money. Accessing a private doctor means I don’t have to relive my traumatic experiences to every doctor in the NHS.* *There’s just been nothing there, nothing to do, no care at all. The care is just so poor at the moment.*	Geoff Emma Debbie
4: Changing care needs in later life – We still need support	*I’m at some sort of crossroads now where I’ve got to decide whether I let my husband buy me a mobility scooter because he gets upset when I’m in pain. Or do I keep taking co-codamol and struggling to try to keep active?* *I’ve really had enough of medication. The side effects of medication for me had been headaches and incontinence, and I haven’t enjoyed either, obviously. So, I didn’t want to go back to headaches and incontinence. I wanted to come off them.* *Now I’m older, the stress of becoming unwell with bipolar has a huge effect on the physical pain I suffer with. People don’t understand that when you are struggling with bipolar and pain, it leads to thoughts of suicide.* *I am starting to experience some forgetfulness, mistakes and so on. It’s beginning to worry me that maybe there’s a connection between bipolar and memory. I want to find out more about it.*	Susan Derek Shirley John

### Part 1: Navigating the Disruption Caused by Diagnosis

Many participants experienced difficulties with their mental health for many years before being diagnosed with bipolar disorder. Often, they were diagnosed with bipolar disorder as younger adults but identified receiving this diagnosis as a critical event in their lives that led to confusion and fear. Certain participants also felt that a lack of support and information at this time exacerbated their concerns. This is apparent in Claire’s story, where she described questioning her identity and what the future held following diagnosis.

**Claire:** When I was told I had bipolar disorder, I visualised a woman who was unkempt, unwashed, uncared for, alone, and homeless. I thought, is that me? So, it was horror in many ways. I needed more information, but it wasn’t given.

Following the initial challenges of diagnosis, participants discussed their experiences of seeking support from services. This was a particularly difficult time for participants who described feeling that their agency and control over their lives had been taken away from them. Angela’s story highlighted a loss of agency following her diagnosis of bipolar disorder.

**Angela:** When I first got the diagnosis, they said you’re going to have to start taking these sodium valproate tablets. I said how long for, and they just said, well, forever probably. I do have a memory of just taking the first one and thinking God, I’m going to be on this forever, and this is me now.

For some participants, their narratives portrayed feelings of hopelessness as they felt their care needs were not considered, their voices were not heard, and that care was ‘done to them’ rather than alongside or in collaboration with them. Karen’s narrative exemplified this, as she discussed her difficult experiences in a psychiatric hospital after receiving a diagnosis of bipolar disorder.

**Karen:** After I was diagnosed and put in the hospital, my needs weren’t addressed at all. Things were just done to me, and I had to put up with it.

Other participants revealed contrasting experiences of being diagnosed with bipolar disorder. Susan stated that this was an important event that provided her with access to psychological support, which helped her to make sense of traumatic experiences leading up to diagnosis. For Susan, this support helped her to improve her understanding of bipolar disorder and develop self-awareness that still helps her to manage mood instability to this day.

**Susan:** The psychologist was understanding, she was respectful, she was humane, kind, patient and also very knowledgeable; she was absolutely superb. She took her time with me listening and letting me cry and eventually said this goes back to early childhood, so we need to go right back to the beginning. It helped me so much and helped me to realise that it wasn’t all my fault. That still helps me today.

However, the majority of participants expressed feeling stigma and rejection at this time. Amy’s story highlighted the pain of feeling judged by family, friends, and society following diagnosis.

**Amy:** Being diagnosed brings pretty awful discrimination, and it’s hard to undo it once you have any kind of label attached to you.

Linda described how her friends questioned her after she stopped working due to bipolar disorder. For Linda, this was a difficult experience that left her feeling isolated and misunderstood.

**Linda:** My friends did not understand because none of them have bipolar disorder. When I stopped working, they used to question me and say well, you’re not ill now, so why are you not going to work? The stigma was appalling, and I just received more and more abuse from everyone.

Overall, participants highlighted that receiving a diagnosis of bipolar disorder was a pivotal moment in their lives. This generally left them experiencing complex emotions and seeking support to integrate bipolar disorder into their lives.

### Part 2: The Removal of Services That Provided Hope

After being diagnosed with bipolar disorder, many participants felt isolated and worried about their future, especially after being discharged from services such as inpatient psychiatric hospitals. A key theme within participants’ narratives was the importance of community care being introduced in the early 1990s. This meant that following discharge from the hospital, participants had an allocated community psychiatric nurse (CPN) who visited consistently and provided emotional and practical support. Participants described the importance of developing consistent, trusting relationships with professionals who understood their care needs, offered compassion, and initiated quick access to support when necessary. Claire described how her CPN was a reliable point of contact during crisis, which provided her with a sense of safety and security whilst living with bipolar disorder that benefitted her wellbeing.

**Claire:** My CPN didn’t come across in any way that he was judging our situation. He was amazing and made me feel safe. They almost became part of the family. It was so helpful and validating for me.

Finding peer support groups was also a powerful moment in participants’ journeys post-diagnosis. Being able to share their experiences with people who understood and accepted them despite experiencing bipolar disorder was described as an important area of support for most participants. This point is demonstrated by David’s narrative, which outlined the importance of being welcomed into a bipolar peer support group following many years of feeling outcast and marginalised.

**David:** I joined a bipolar support group when I moved towns, and it was run by someone with bipolar. It was an astonishing feeling to be accepted; it was brilliant.

However, participants’ narratives often changed to a more pessimistic tone as they began to age. Susan described the devastating impact of learning that resource scarcity would leave her without CPNs or community care.

**Susan:** When my CPN retired, I was bereft; I was so sad. I didn’t know what I was going to do if something went wrong. Since then, I’ve had nothing; I have been on my own and unable to get any help outside of care when I’m in crisis.

Participants’ narratives highlighted a sense of loss as they felt isolated and abandoned by services and without access to care that would meet their needs. This change in narrative tone was portrayed in Mandy’s story where she highlighted the sense of loss when her CPN was taken away.

**Mandy:** It was great, but then they started saying you’re only getting a CPN if you’re in services, and the minute you’re out of services, you’ve got to go through the whole process again, and there’s no guarantee you’ll get the same one. It was much less organised, and the service cuts hit me hardest when I lost the CPN who kept me going between crises. After that, there was nothing.

These difficulties were often compounded when a number of participants reported that the peer support groups were shut down without consultation with themselves or other group members. For many participants, peer support groups were a safe, supportive space, and their removal was frustrating and isolating.

**Emma:** It was so helpful to see how other people with bipolar disorder live, and somehow the group I went to closed down; we don’t know why, and no one ever explained why, which is very bad; it is patronising. It is so frustrating because it leaves us very isolated.

Whilst the majority of participants’ narratives portrayed a sense of loss and frustration as they aged, one participant had maintained contact with a long-term psychiatric nurse. Karen highlighted how this continuity of care enabled her to maintain stability in later life by helping her to reduce isolation and increasing her motivation to stay well. This support also provided security as she entered later life with bipolar disorder, showing the power of this relationship and the value of consistent care for older adults with bipolar disorder.

**Karen:** Having a psychiatric nurse whom I’ve known for years is like a friend visiting. It provides so much security, and I’d be lost without her.

Participants’ narratives illustrated the importance of being able to access care that was person-centred and compassionate and allowed them to develop meaningful relationships with staff. This theme also highlighted the sense of loss that participants felt when this care was taken away and the frustration that followed.

### Part 3: Later Life – We Are on Our Own Now

As participants’ narratives moved into later life, a common theme was that there was a substantial gap where they had no contact or support from services. Many felt all essential support was now inaccessible or had been removed, leaving them feeling disappointed and increasingly alienated from services. Mike’s narrative highlighted his disappointment at the support offered by services within the United Kingdom at present.

**Mike:** Britain has the problem that the medical fraternity seems to have dropped the idea of care. There is no care anymore. It’s just about medication delivery now. It is so frustrating and unhelpful.

Participants stated that they had learnt to adapt and manage bipolar disorder alone or alongside family as they aged. They explained that learning to cope in this way made them highly self-reliant in later life. However, Emma described how her experiences of learning to cope with bipolar disorder and her frustration at being unable to access services led to her rejecting services altogether as she aged.

**Emma:** No, I don’t want support from services now. I managed to sort everything myself. Basically, everything that I needed was not given. So eventually, you become very self-reliant, and you just cope with the help of family.

Other participants described frustration and confusion as they sought support as older adults. For example, participants still desired help from services to provide reassurance but were left exasperated by their inability to access appropriate care in later life. This was a common experience, and Linda’s narrative outlined how she only realised that she had been discharged from services when trying to access urgent support during crisis. This left her feeling that a vital safety net had been taken away without her knowledge, leaving her feeling isolated and alone.

**Linda:** I rang and said I’m stressed and need to speak to somebody. They said your case isn’t active anymore, so why are you ringing us? It was only two years ago that I rang when I needed help, and now I can’t access help.

While these issues were observed in most participants in later life, other participants had contrasting experiences. For Mike, although he had experienced challenges with services throughout his life, he reported that his recent experiences of accessing support from the older adult community mental health team were resoundingly positive. Mike outlined the benefits of this support as an older adult, as it provided a safety net that was missing for other participants, such as Linda. However, Mike’s narrative highlighted how he had to fight to get support from services for many years before he finally received adequate care and support as an older adult.

**Mike:** I was recently referred to an older adult mental health team, which was very positive as they responded quickly and supported me. I couldn’t have expected more. It was just that reassurance that the safety net was there. It was such a relief, but I’ve had to fight for years to get this, and it’s been exhausting.

In summary, this theme demonstrated the challenges participants experienced when attempting to access appropriate care in later life. The challenges associated with this led to participants disengaging with services and attempting to cope alone. However, participants who were able to access appropriate support expressed a positive impact on their wellbeing, indicating the importance of offering appropriate care for this group.

### Part 4: Changing Care Needs in Later Life – We Still Need Support

Part 4 of the participants’ narratives highlighted how they felt their care needs had changed as they aged and that despite disengaging with services to avoid stress and frustration, they would still like support if it was more readily accessible. In particular, participants felt worried that they now had to manage the physical side effects caused by living with bipolar disorder for many years and wanted support. For example, Claire voiced concerns over her physical health after using medication to manage mood instability for several years.

**Claire:** Antipsychotics are known to have cardiac impacts. I’ve been on antipsychotics for a very long time, and I’m worried about what that could do. So, I worry more about the physical side of things now rather than being carried off by a wave of emotions like in the past.

Angela’s story further highlighted participants’ concerns over the long-term physical health impact of taking mood-stabilising medication for bipolar disorder. Angela’s narrative illuminated how she had accepted medication as part of her life but suggested that she would like more information about its potential consequences in later life. However, this information was still difficult to access from professionals.

**Angela:** I’ve had to accept that taking a mood stabiliser is part of my life. For years, I have asked what sodium valproate does to your body over the long term, but nobody has ever given me much of an answer to that one. From doing my own research, it seems like it does have various physical health side effects, which is concerning. I would like more information, but it is not available.

Participants displayed how becoming older had brought additional financial stressors. When younger, participants placed emphasis on achieving success within their careers; however, many felt that this had contributed to significant stress and multiple mood episodes throughout their lives. As older adults, several participants wanted to retire or reduce their workload to reduce the risk of relapse. Susan’s story showed the challenges of managing the trade-off between retiring to reduce stress, surviving financially, and engaging in activities that brought her fulfilment and helped her to maintain stability as she aged.

**Susan:** How much do I give in? Our lives have had to shrink and will continue to shrink because of finances. Next year, there will be a big drop in income, and I could be migrated to universal credit, which would be horrible. At the moment, I can afford to do things I enjoy, but I’m worried about what the future holds if I can’t do that.

Finally, many participants suggested that a key care need in later life was to be able to develop meaningful connections with professionals, as this made them feel safe, supported, and understood. This was apparent in Geoff’s narrative, where developing a meaningful relationship with a psychiatrist helped him to feel understood, safe, and supported which benefitted his wellbeing and ability to cope with challenges in later life.

**Geoff:** I have been really blessed to have a great psychiatrist in my life. We got on from the moment I walked into the room. Having that relationship with him where I feel comfortable with him has helped me massively. It helps me to cope when things get difficult.

However, participants were frustrated that many services now operate via remote methods, such as using the telephone or online appointments. The use of technology was not only daunting for some participants but also limited their ability to engage in human contact, which seemed beneficial. For Linda, her inability to access face-to-face support and establish meaningful connections with professionals had a negative impact on her wellbeing.

**Linda:** You need to be able to talk to a human. I need a person I can talk to when the moment hits. I’ve found that as I’ve got older, there isn’t any human to contact anymore. It’s a complete nightmare.

Whilst many participants reported difficulties in accessing care that met their changing care needs in later life, one participant’s narrative highlighted the personal impact of accessing effective care as she aged with bipolar disorder. Shirley’s account demonstrated the value of accessing support for her physical and mental health needs from services and family. She described how feeling supported, respected, and valued in later life made life worth living again despite the challenges posed by ageing with bipolar disorder. Before receiving this support, Shirley discussed how she often felt unable to cope and contemplated suicide due to physical pain caused by arthritis and mental health challenges associated with bipolar disorder.

**Shirley:** I can’t argue with the care I’ve had in terms of psychology and help with physical health. It’s been fantastic as I’ve got older. Having the right support in place from services and family who understand can make life worth living again.

This theme highlighted that the care needs of older adults with bipolar disorder change in later life. As a result of this, participants argued that care requires adaptations to meet their needs and support them to manage the transition into later life whilst living with bipolar disorder.

## Discussion

This study contributes to existing literature by capturing the changing care and care needs of older adults with bipolar disorder in later life. Participants initially described their confusion and fear after being diagnosed with bipolar disorder and their attempts to navigate through this period whilst experiencing stigma and judgement from others. Narratives then highlighted a brief period of hope where community care and peer support groups were readily accessible before the removal of these services caused stress and frustration for participants. As participants transitioned into later life, many felt that they were ‘on their own’ and unable to access appropriate services. This led to participants disengaging with services completely at times to avoid iatrogenic stress and attempting to cope alone or alongside family. Finally, older adults with bipolar disorder in this study expressed how their care needs had now changed due to concerns over their physical health, managing finances as they retire, and difficulties in accessing face-to-face care where they can develop meaningful relationships with professionals. Whilst some participants were still able to access care and described the benefits of this, this study generally illuminated a disconnect between the care this group currently received and the care they desired. It is possible that this contributes to the range of challenges and poor outcomes commonly observed in older adults with bipolar disorder.

Consistent with the life course perspective (Dannefer & Settersten, 2010), the inequalities associated with bipolar disorder throughout participants’ lives appeared to lead to challenging consequences as they aged. Older adults with bipolar disorder experienced unique care needs, including concerns surrounding the physical health consequences of using mood-stabilising medication over several years. Existing literature suggests that older adults with bipolar disorder may be at increased risk of physical health comorbidities such as cardiovascular disease and certain forms of cancer (Warner et al., 2023). Consequently, services should attempt to address the potential side effects of long-term medication use and other physical health challenges associated with ageing with bipolar disorder (Dols et al., 2013). Alongside this, participants felt that retirement would help to reduce stress and protect their mental health but were apprehensive about the associated financial challenges. Literature from the general population suggests that retirement can increase the risk of mental health challenges and mobility issues (Dave et al., 2008). Our results indicate that treatments for older adults with bipolar disorder should also prioritise practical advice surrounding the transition into retirement and financial support that enables this group to live well as they age. Despite this, NICE guidelines still recommend similar treatments to younger age groups with bipolar disorder, meaning the care older adults with bipolar disorder receive may continue to be insufficient to meet their different needs (NICE, 2014).

Participants spoke openly about the benefits of receiving community care in the past and their disappointment at no longer being able to access this support. The Community Care Act was introduced within the NHS in 1990 with the aim of offering practical and high-quality care that supports people to live within their own homes wherever sensible and feasible (Thornicroft, 1994). However, cuts within NHS services, lack of staffing, and reduced funding, alongside increased demand for services as people continue to live longer, have led to severe challenges in accessing community care (Howse, 2008). Furthermore, older adults who often require the most support from services as they experience complex comorbidities are at risk of becoming isolated and neglected (Howse, 2008). Our findings suggest that older adults with bipolar disorder now often attempt to manage difficulties alone or with support from family as they struggle to access care that meets their changing needs. These findings are in contrast with the NHS Long Term Plan and NHS Mental Health Implementation Plan (NHS England, 2019), which outlines that a key aim is to increase access to community care for older adults with mental illness and ensure that no underlying need is missed. Additionally, participants highlighted their frustration at being unable to access face-to-face appointments with professionals and emphasised that the stress caused by this led to disengagement. These findings contradict those of a recent review demonstrating that using technology for mental healthcare delivery leads to improved health outcomes, improved cognitive function, and reduced symptoms in older adults experiencing depression (Harerimana et al., 2019).

Peer support has been found to be beneficial for people experiencing mental health difficulties and has been linked to reduced admission rates, improved clinical outcomes, and reduced stigma (Mahlke et al., 2014; Repper & Carter, 2011). Our analysis suggests that peer support can be particularly important for older adults with mental health difficulties as they often experience a dual stigma that results in disengagement from services and poor quality of life (Depla et al., 2005). Participants’ narratives supported this and demonstrated the benefits of acceptance from peers after experiencing continued stigma and rejection as they aged. Narratives highlighted that peer support groups were often closed without consulting participants or other group members, leaving older adults with bipolar disorder unable to access peer support. This contributed to isolation and loneliness, which have been found to be the strongest predictors of mental health outcomes in older adults (Donizzetti & Lagacé, 2022). To avoid the removal of services that appear to benefit older adults with bipolar disorder, services should actively collaborate with this group to ensure that the support offered is tailored to their needs. Research and policy have identified the importance of service user involvement when designing care (Wright et al., 2016) and using the views of older adults with bipolar disorder to inform service adaptations can help to improve care and reduce the challenges discussed within this study.

## Strengths and Limitations

There are several strengths in this study. First, the authors utilised biographical narrative interviewing methods that allowed them to capture in-depth stories of participants’ experiences of care throughout their lifespan and identify their changing care needs as they transition into later life (Crossley, 2000). Although the sample size was small, it allowed the researchers to capture in-depth stories that helped to address the research aims as comprehensively as possible (Smith, 2016). This helps to build upon the findings of existing literature (Sajatovic et al., 2015) and make sense of why older adults with bipolar disorder experience unique challenges requiring treatments to be adapted to reduce the difficulties they experience. Additionally, the authors ensured that patient and public involvement was incorporated at all stages of the research study, therefore enhancing reflexivity, improving rigour, and highlighting researcher bias which is essential when completing qualitative research (Morse, 2015).

Limitations include a small convenience sample, which included few participants currently receiving care from NHS services. Many had been discharged or struggled to access NHS or other services, which may have led to more negative accounts of services. However, the failure to recruit more participants actively receiving NHS care may also reflect current conditions within the NHS, where staff shortages result in staff having to prioritise high-risk cases and clinical care rather than supporting research participation (Clarkson et al., 2023). Participants in this study were predominantly educated, White British, and retired professionals. Consequently, the experiences of care and support and the care needs of this group may not be representative of all older adults living with bipolar disorder.

## Future Research Directions

Future research should aim to build on the findings of this study by investigating ways to improve support for older adults with bipolar disorder. Participants expressed concerns surrounding the long-term physical consequences of using mood-stabilising medication for many years. This link should be investigated in more depth as it may have vital implications for care. Older adults with bipolar disorder also expressed feeling unable to access appropriate services as they aged or had completely disengaged with services in later life to avoid the risk of further iatrogenic harm. Research that aims to uncover the barriers faced by older adults with bipolar disorder as they attempt to access care, what leads to disengagement and harm, and how to reduce these barriers would be beneficial. Work such as this may help to instil hope in older adults with bipolar disorder, improve their trust in services, and enhance their engagement with services moving forward.

## Conclusion

This study highlighted that the care needs of older adults with bipolar disorder change significantly over time and particularly in later life. Key concerns include the side effects of long-term medication use, barriers to accessing care, and frustration at the removal of potentially beneficial services as they age. Services should develop an ongoing collaborative partnership with older adults with bipolar disorder and work with them to adapt services to meet their changing individual care needs. This collaborative and person-centred approach will improve the support available to older adults with bipolar disorder, increase engagement, and enhance their wellbeing, enabling them to live fulfilling lives as they age.
